# Morgagni-Larrey Hernia: A Possible Cause of Recurrent Lower Respiratory Tract Infections

**DOI:** 10.7759/cureus.4035

**Published:** 2019-02-07

**Authors:** Muhammad Usman Hashmi, Kaleem Ullah, Ayesha Tariq, Mohsin Sarwar, Iftikhar H Khan

**Affiliations:** 1 Surgery, Shifa College of Medicine, Islamabad, PAK; 2 Thoracic Surgery, Nishtar Medical University Hospital, Multan, PAK; 3 Internal Medicine, Shifa College of Medicine, Islamabad, PAK

**Keywords:** morgagni hernia, omentum, congenital diaphragmatic hernia, larrey hernia, sternocostal triangle, recurrent respiratory tract infections

## Abstract

Morgagni-Larrey hernia is an exceedingly rare presentation of congenital diaphragmatic hernia. Despite its rarity, it is associated with significant risk of morbidity and mortality. Herein, we describe a unique case report of an elderly woman who presented with left-sided chest pain, dyspnea, and chronic history of recurrent respiratory tract infections. On the basis of her medical history, general physical examination and imaging studies, she was operated for a presumptive diagnosis of thymolipoma. However, the intra-operative findings revealed that it was an unusual variant of a diaphragmatic hernia and the hernia sac appeared through the retrosternal foramen of Morgagni. Hence we concluded that it was a Morgagni-Larrey hernia compressing the lungs and heart. Consequently, the hernia was reduced and the defect was repaired. During the postoperative period, the patient had an uneventful recovery. To conclude, the possibility of a Morgagni-Larrey hernia should be strongly considered while evaluating a patient with recurrent chest infections, dyspnea, and vague chest pain.

## Introduction

Foramen of Morgagni is a triangular space in the anterior thoracic wall. It is located between the muscular fibers of the sternal and costal attachments of the diaphragm. This potential space for the development of a hernia lies just posterolateral to the sternum at the level of the seventh rib on both sides of the xiphisternum [1]. Under normal conditions, this space is filled with a variable amount of fat. Furthermore, a pleural layer covers it superiorly, whereas the peritoneum lines it from below. In the case of a Morgagni hernia, the peritoneum and the contents of the abdomen herniate through this potential weak area into the thoracic cavity [2]. They are more often right sided (90%), smaller in size, and rarest among all diaphragmatic hernias as they constitute just 3% of all the diaphragmatic hernias [3]. The Morgagni-Larrey hernia sac mostly contains the transverse colon with the omental fat. Rarely, the stomach, loops of small intestine, and liver can be present in the hernia sac [4]. As far as the clinical presentation is concerned, it is usually asymptomatic in the adult population and is mostly diagnosed incidentally from a chest X-ray performed for other etiologies of respiratory symptoms. However, some cases may have dyspnea, cough, chest pain, and obstructive symptoms. In this case report, we describe a patient who was suffering from recurrent respiratory tract infections for many years [5]. This condition remained undiagnosed for a very long period. Therefore, we are presenting this case to raise awareness among healthcare professionals regarding this rare clinical presentation of a Morgagni-Larrey Hernia. The authors hope that this study will help the junior doctors, especially surgical trainees to consider this in the differential for early diagnosis and prevention of life-threatening complications of this clinical entity.

## Case presentation

**Figure 1:**
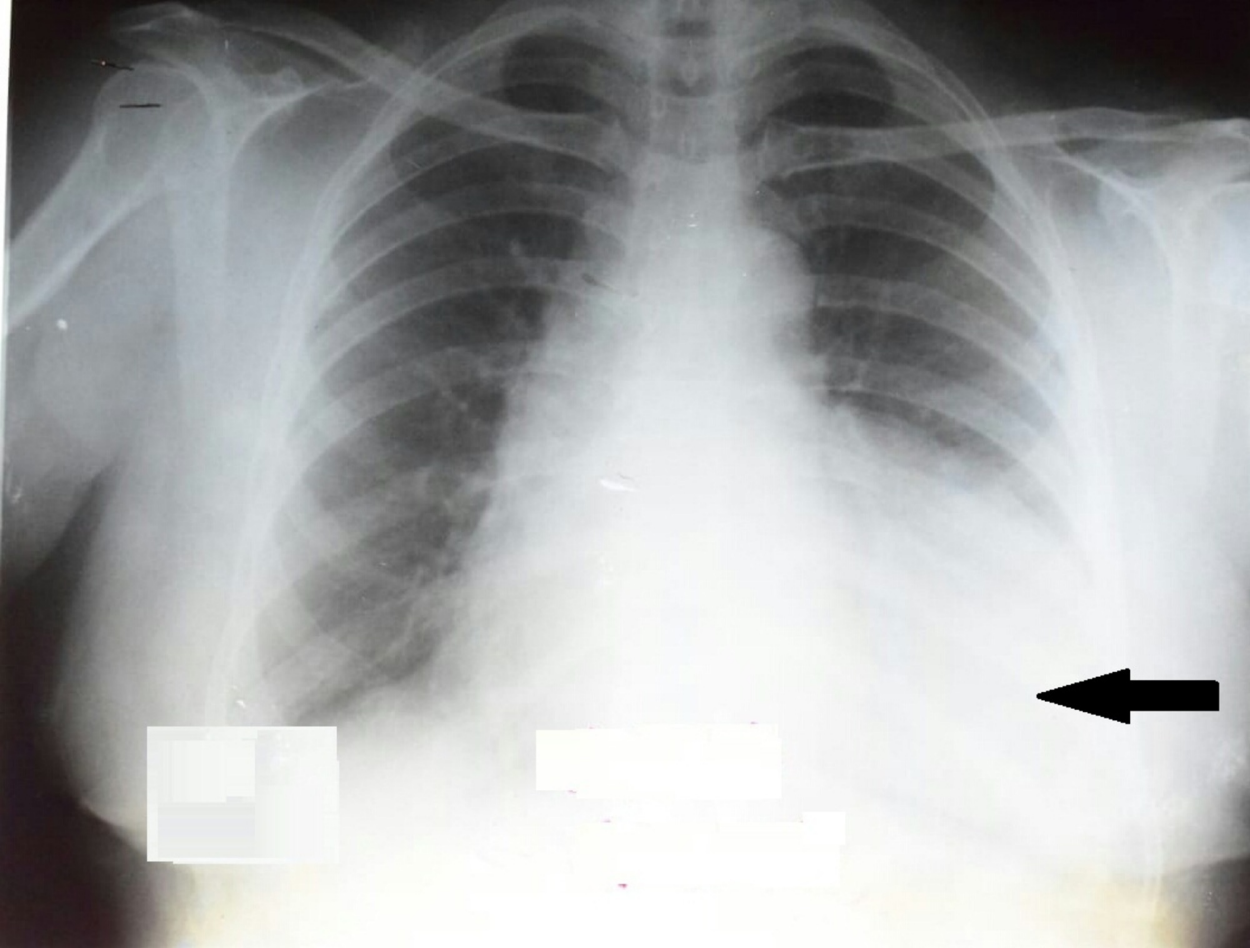
Posteroanterior chest radiograph showing a non-homogeneous opacity involving the left lingual segment and left lung lower lobe (marked by an arrow). Loss of silhouetting of the left heart border is also evident.

A 57-years-old female presented with complaints of recurrent chest infections. She also complained of dull, aching left-sided chest pain associated with dyspnea and orthopnea. She had no complaints of palpitations, exertional dyspnea, heartburn, jaundice, nausea, and vomiting. There was no history of a change in bowel habits and occurrence of abdominal pain. However, the patient narrated a longstanding history of recurrent lower respiratory tract infections associated with a productive cough and fever. She would seek medical care from a local doctor who used to treat her illness conservatively by prescribing antibiotics. Her symptoms usually resolved over a couple of months. The maximum interval between consecutive episodes was three months. Keeping in view her cumbersome condition, she was referred to the Nishtar Medical University Hospital in Multan, Pakistan for further evaluation and management. The patient was thoroughly re-assessed. On general physical examination, the patient was hemodynamically stable with a pulse rate of 88 beats per minute, blood pressure of 130/85 mmHg and an oral temperature of 39°C. Chest movements and breath sounds were decreased on the left side with a dull percussion noted when compared to the right side. There were no added sounds. Chest X-ray was ordered which revealed a non-homogenous opacity involving the left lingual segment and left lung lower lobe with loss of silhouetting of the left heart border. Moreover, there was a mild mediastinal shift to the right side along with a positive hilum overlay sign. However, the cardiac borders were not well appreciated. Figure 1 shows the plain chest radiograph of this patient. 

**Figure 2:**
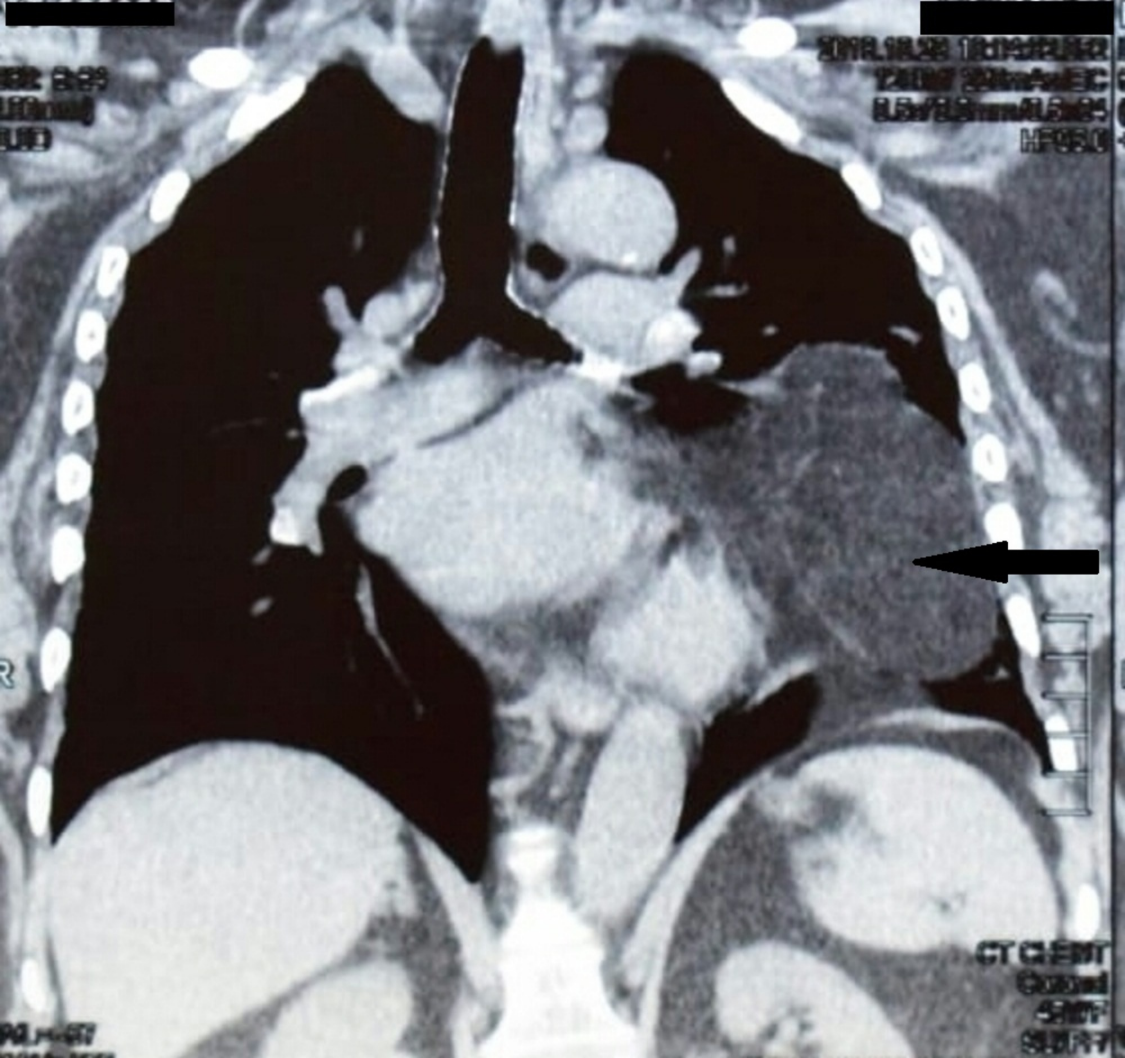
Sagittal view of chest computed tomography scan revealing a large fat density mass arising from the anterior mediastinum and extending along the left pericardium occupying half of the left hemithorax (marked with an arrow).

Echocardiography was also performed showing concentric left ventricular hypertrophy. There was no evidence of pericardial effusion with an ejection fraction of 60%. Chest computed tomography (CT) scan revealed a large fat density mass with internal linear strands of soft tissue arising from the anterior mediastinum and extending along the left pericardium occupying half of the left hemothorax. The mass was displacing and compressing the ipsilateral lung resulting in atelectasis of adjacent lung parenchyma and contralateral mediastinal shift. Figure 2 shows the chest CT scan of this patient.

**Figure 3:**
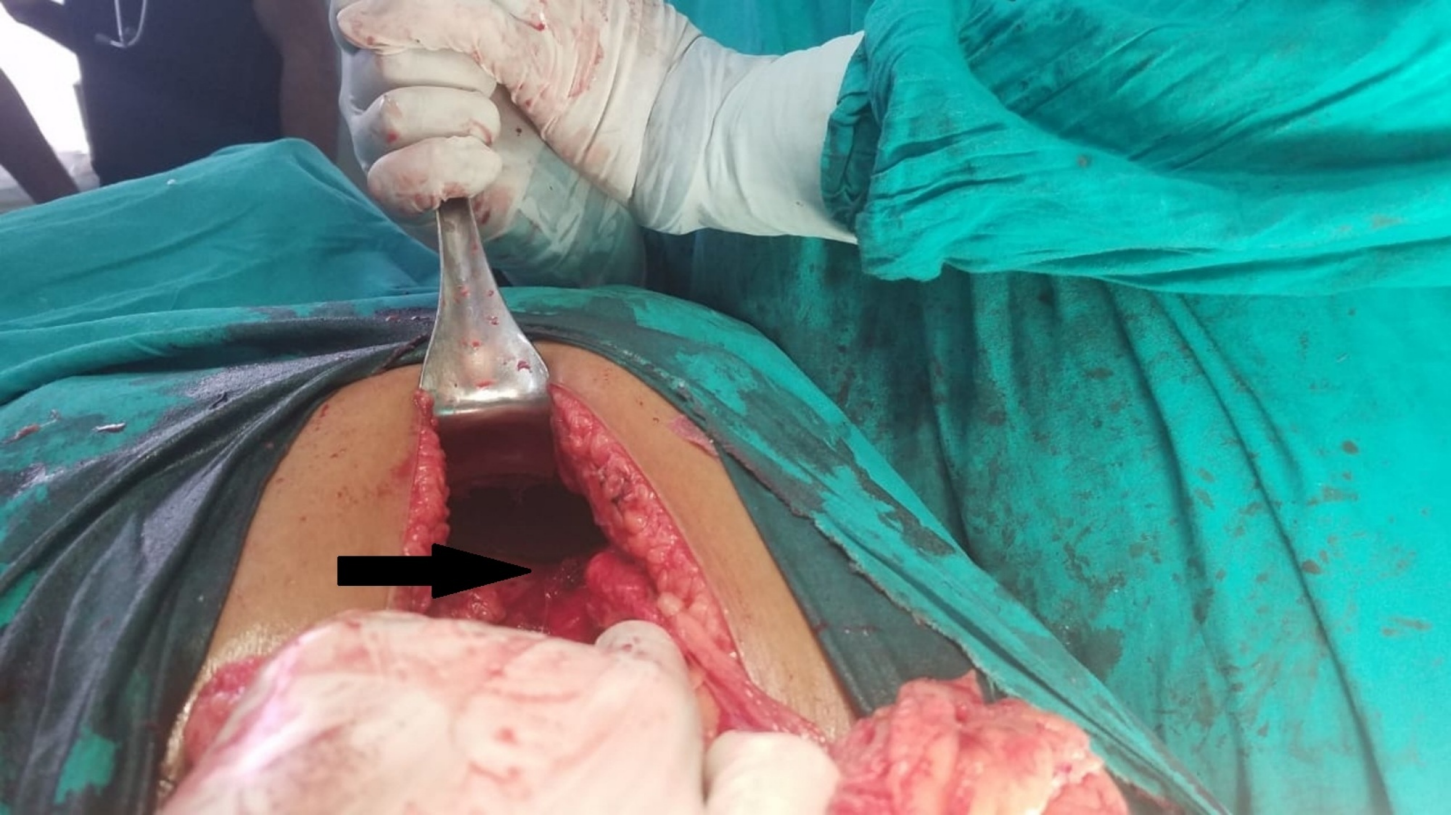
The black arrow indicates the defect in the diaphragm, the site of the Morgagni-Larrey hernia.

A presumptive diagnosis of thymolipoma was made. Consequently, left-sided postero-lateral thoracotomy was performed. Intraoperatively, a huge globular mass was passing through a rent in the diaphragm measuring about 8 cm. It was situated at the medial end of the xiphoid process near the attachment of central tendon. This large defect in the diaphragm is shown in Figure 3.

**Figure 4:**
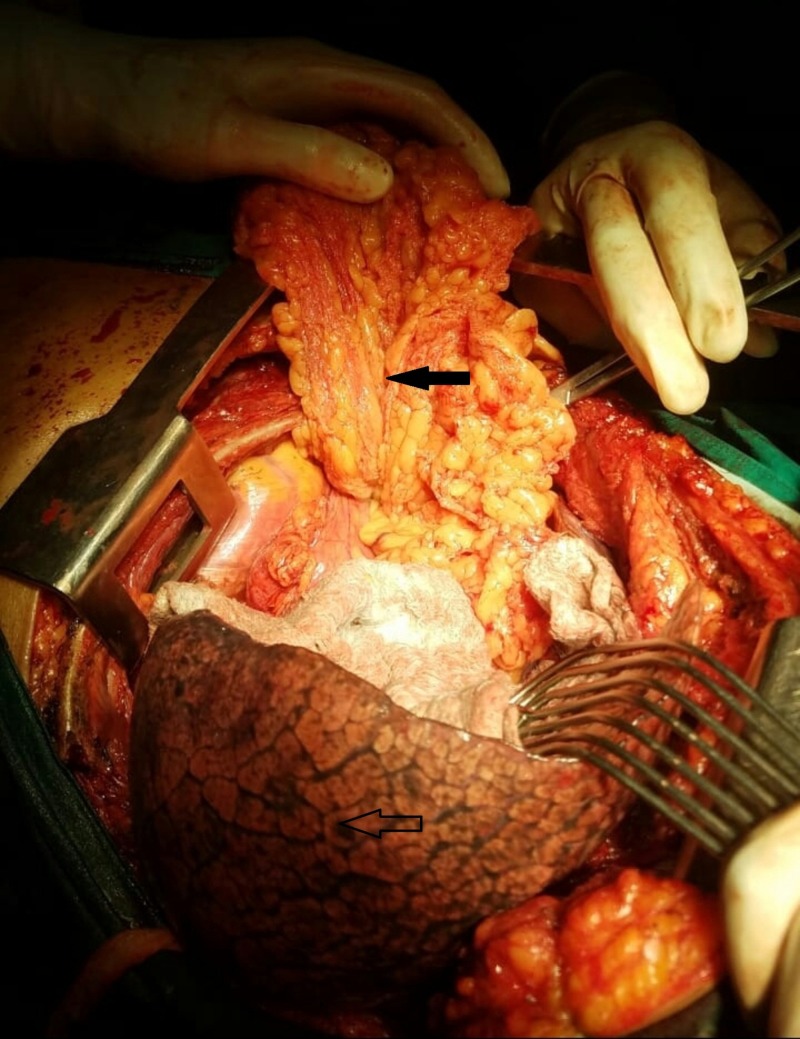
Intraoperative view of the Morgagni-Larrey hernia repair. The large hernial sac containing omentum within it was present in the thoracic cavity (marked by solid black arrow). The hernial sac was also compressing the adjacent lung parenchyma (marked by unfilled arrow).

The hernia sac passed anteromedial to the heart and great vessels and was pushing into the pleural cavity. Intraoperative findings are shown in Figure 4.

Following the confirmation of the findings, the thoracic cavity was closed after placing a left-sided chest drain. Thereafter, an upper midline laparotomy was performed to reduce the omentum back into the abdomen. The defect in the diaphragm was repaired using a synthetic polypropylene mesh. The patient had an uneventful recovery. She was discharged on the sixth postoperative day and was followed up at the intervals of two weeks, two months, and six months. During all these visits, the patient remained asymptomatic and there was no associated recurrence.

## Discussion

Morgagni-Larrey hernia is a rare clinical entity which was first described by an Italian anatomist Giovanni Battista Morgagni [6]. The review of the medical literature reveals that there is confusion in the nomenclature of this type of a hernia. Studies have used the term Larrey's hernia to describe this unusual type of hernia if it occurs on the left side of the sternum, while Morgagni hernia referred to a right-sided hernia [7]. To eliminate this confusion, it should be recalled that Larrey never described a hernia. Rather, he described that the sternocostal triangle (Larrey’s space) can be used as a surgical approach to drain cardiac tamponade [8]. Therefore, it is suggested that any anterior diaphragmatic hernia, either right or left, should be named as the hernia of Morgagni, or Morgagni-Larrey hernia [2].

Most cases are diagnosed late because of the vague respiratory or upper gastrointestinal symptoms. The clinical presentation of our case was quite misleading, as the patient had recurrent lower respiratory tract infections due to the presence of Morgagni-Larrey hernia. Therefore, her indistinct symptoms of the respiratory system caused further delay in the establishment of the diagnosis. She got treatment for lower respiratory tract infections with no clue of clear etiology. It is important to note that it took many years to correctly diagnose the problem of our patient. Moreover, this condition severely affected the patient’s physical and mental health. It compromised her quality of life. At times, she was unable to perform her daily routine activities due to this prolonged morbid condition. This highlights the significance of an early diagnosis of patients presenting with such symptomatology. Therefore, the authors highly recommend that while evaluating a patient with recurrent respiratory tract infections, a thorough abdominal examination and, if required, radiological evaluation should be performed to exclude this potentially life-threatening clinical entity.

Another point we want to put forward is the significance of a thorough preoperative workup. An optimal preoperative workup can help to evaluate the patient and to prevent any missed diagnosis. For instance, if omentum is present in the sac, chest X-ray will show a paracardiac non-homogenous opacity with loss of silhouetting of the heart borders. The differential diagnosis includes atelectasis, intrathoracic tumor, pneumonia, a pericardial cyst or Morgagni-Larrey hernia. In this scenario, barium studies can help to differentiate this type of a hernia from other causes. When the hernia sac contains gastrointestinal loops, the diagnosis is clear. Even in cases where omentum is in the sac, a part of the gut or stomach is also present or is pulled up with the omentum causing its distortion and points towards the diagnosis of Morgagni-Larrey hernia. However, in some cases, barium contrast studies performed for gastrointestinal symptoms can also be absolutely normal [7]. Therefore, the use of CT scan is advocated as an excellent, accurate, and non-invasive method of diagnosing Morgagni-Larrey hernia [4].

In cases of Morgagni-Larrey hernia, surgical treatment option is advised due to the high incidence of complications such as strangulation, obstruction, or incarceration [4]. When the diagnosis is certain, the transabdominal approach is preferred as it allows easier reduction of a hernia. Moreover, abdominal viscera present within the hernia sac can be pulled down to their normal location in the abdomen. In our patient, we opted for an abdominal approach and closed the defect in the diaphragm with polypropylene mesh as the rent was significantly large. The review of the medical literature reveals that surgery can be performed via laparotomy or a minimally invasive approach [8-9].

## Conclusions

Morgagni-Larrey hernia is a rarely encountered clinical entity, which can be the cause of recurrent lower respiratory tract infections. Therefore, this case report highlights that the possibility of Morgagni-Larrey hernia should be kept in mind when a patient presents with a chronic history of recurrent chest infections along with vague chest pain and dyspnea.

## References

[1] Bordoni B, Zanier E (2013). Anatomic connections of the diaphragm: influence of respiration on the body system. J Multidiscip Healthc.

[2] Paris F, Tarazona V, Casillas M, Blasco E, Cantó A, Pastor J, Acosta A (1973). Hernia of Morgagni. Thorax.

[3] Griffiths EA, Ellis A, Mohamed A, Tam E, Ball CS (2019). Surgical treatment of a Morgagni hernia causing intermittent gastric outlet obstruction. BMJ Case Rep.

[4] Minneci PC, Deans KJ, Kim P, Mathisen DJ (2004). Foramen of Morgagni hernia: changes in diagnosis and treatment. Ann Thorac Surg.

[5] Aghajanzadeh M, Khadem S, Jahromi SK, Gorabi HE, Ebrahimi H, Maafi AA (2012). Clinical presentation and operative repair of Morgagni hernia. Interact Cardiovasc Thorac Surg.

[6] McBride CA, Beasley SW (2008). Morgagni's hernia: believing is seeing. ANZ J Surg.

[7] Rodríguez Hermosa JI, Rodríguez FT, Feliu BR (2003). Diaphragmatic hernia of Morgagni-Larrey in adults: analysis of 10 cases [Article in English, Spanish]. Gastroenterol Hepatol.

[8] Rau HG, Schardey HM, Lange V (1994). Laparoscopic repair of a Morgagni hernia. Surg Endosc.

[9] Young MC, Saddoughi SA, Aho JM (2019). Comparison of laparoscopic versus open surgical management of Morgagni hernia. Ann Thorac Surg.

